# Supplementary material for: Evolutionary sparse learning with paired species contrast reveals the shared genetic basis of convergent traits
Source: bioRxiv. 2025 Jan 8:2025.01.08.631987. Preprint. [Version 1] doi: 10.1101/2025.01.08.631987 (PMC11741315; doi:10.1101/2025.01.08.631987)
Supplement: Supplement 1 [file NIHPP2025.01.08.631987v1-supplement-1.pdf]

bioRxiv preprint doi: https://doi.org/10.1101/2025.01.08.631987; this version posted January 9, 2025. The copyright holder for this preprint (which was not certified by peer review) is the author/funder, who has granted bioRxiv a license to display the preprint in perpetuity. It is made available under aCC-BY 4.0 International license.

Extended data

Supplementary Table 1: Echolocation ensemble model top genes

Rank	Gene identifier	Ensembl accession number	GSS	# combos ranked in top 1%
1	CASQ1	ENSG00000143318	0.1659	16
2	TMC1	ENSG00000165091	0.114	16
3	ADAMTS1	ENSG00000154734	0.0766	16
4	CDH23	ENSG00000107736	0.0713	16
5	CELA1	ENSG00000139610	0.1366	16
6	GSN	ENSG00000148180	0.0628	16
7	PAH	ENSG00000171759	0.0613	16
8	TBC1D14	ENSG00000132405	0.0418	16
9	SLC26A5	ENSG00000170615	0.097	16
10	GIGYF2	ENSG00000204120	0.0914	16
11	NDRG2	ENSG00000165795	0.0694	16
12	EPYC	ENSG00000083782	0.0847	16
13	ODF1	ENSG00000155087	0.0625	16
14	HORMAD2	ENSG00000176635	0.0312	16
15	TBC1D17	ENSG00000104946	0.061	16
16	RTN4RL2	ENSG00000186907	0.0591	16
17	RIC3	ENSG00000166405	0.0514	16
18	PTCH2	ENSG00000117425	0.036	16
19	LINGO2	ENSG00000174482	0.0532	16
20	BRINP2	ENSG00000198797	0.0525	16
21	CCR8	ENSG00000179934	0.0238	16

bioRxiv preprint doi: https://doi.org/10.1101/2025.01.08.631987; this version posted January 9, 2025. The copyright holder for this preprint (which was not certified by peer review) is the author/funder, who has granted bioRxiv a license to display the preprint in perpetuity. It is made available under aCC-BY 4.0 International license.

22	DUSP2	ENSG00000158050	0.0494	16
23	EML5	ENSG00000165521	0.0236	16
24	PTBP1	ENSG00000011304	0.0514	16
25	GFM1	ENSG00000168827	0.0359	16
26	CHD1L	ENSG00000131778	0.0186	16
27	HORMAD1	ENSG00000143452	0.0469	16
28	DHX16	ENSG00000204560	0.0474	16
29	SRRM4	ENSG00000139767	0.0202	16
30	NUDCD1	ENSG00000120526	0.0294	16
31	ELOVL7	ENSG00000164181	0.0345	16
32	PHB2	ENSG00000215021	0.0437	16
33	PNPLA5	ENSG00000100341	0.0166	16
34	RHO	ENSG00000163914	0.0402	16
35	SLC38A2	ENSG00000134294	0.0217	16
36	CABP2	ENSG00000167791	0.0402	16
37	MYO6	ENSG00000196586	0.0298	16
38	RAB22A	ENSG00000124209	0.037	16
39	DDX1	ENSG00000079785	0.029	16
40	VBP1	ENSG00000155959	0.037	16
41	LPGAT1	ENSG00000123684	0.027	16
42	ARHGAP36	ENSG00000147256	0.0159	16
43	MKL1	ENSG00000196588	0.0184	16
44	PTGS1	ENSG00000095303	0.013	16
45	CHRNA9	ENSG00000174343	0.0195	16
46	MARCH6	ENSG00000145495	0.019	16

bioRxiv preprint doi: https://doi.org/10.1101/2025.01.08.631987; this version posted January 9, 2025. The copyright holder for this preprint (which was not certified by peer review) is the author/funder, who has granted bioRxiv a license to display the preprint in perpetuity. It is made available under aCC-BY 4.0 International license.

47	INTS6L	ENSG00000165359	0.0165	16
48	IRF9	ENSG00000213928	0.0115	16
49	VTA1	ENSG00000009844	0.0366	15
50	MAGEB18	ENSG00000176774	0.0191	15
51	SEMA6A	ENSG00000092421	0.0209	15
52	FAM117A	ENSG00000121104	0.0701	14
53	PECR	ENSG00000115425	0.0242	14
54	ATG7	ENSG00000197548	0.0305	14
55	ENPP7	ENSG00000182156	0.0156	14
56	PSEN2	ENSG00000143801	0.0227	14
57	PJVK	ENSG00000204311	0.0208	14
58	PER1	ENSG00000179094	0.0225	13
59	PHF20L1	ENSG00000129292	0.0274	13
60	HSPA12A	ENSG00000165868	0.048	13
61	FAM170A	ENSG00000164334	0.0283	13
62	TNS1	ENSG00000079308	0.015	13
63	LOXHD1	ENSG00000167210	0.0206	13
64	NMUR1	ENSG00000171596	0.0131	13
65	COQ9	ENSG00000088682	0.0218	13
66	YARS	ENSG00000134684	0.0241	13
67	VSIG8	ENSG00000243284	0.0204	13
68	CCSER1	ENSG00000184305	0.0174	13
69	EYA3	ENSG00000158161	0.0514	12
70	MREG	ENSG00000118242	0.0445	12
71	DTX2	ENSG00000091073	0.0307	12

bioRxiv preprint doi: https://doi.org/10.1101/2025.01.08.631987; this version posted January 9, 2025. The copyright holder for this preprint (which was not certified by peer review) is the author/funder, who has granted bioRxiv a license to display the preprint in perpetuity. It is made available under aCC-BY 4.0 International license.

72	PCYT2	ENSG00000185813	0.0411	12
73	SYNC	ENSG00000162520	0.0344	12
74	SPEF1	ENSG00000101222	0.0272	12
75	NKPD1	ENSG00000179846	0.0233	12
76	SEMA5A	ENSG00000112902	0.0155	12
77	THEM5	ENSG00000196407	0.0149	12
78	PEX11G	ENSG00000104883	0.017	12
79	MALT1	ENSG00000172175	0.061	11
80	OTUD3	ENSG00000169914	0.0586	11
81	DGKH	ENSG00000102780	0.028	11
82	HDLBP	ENSG00000115677	0.0275	11
83	GRXCR2	ENSG00000204928	0.0402	11
84	PIGQ	ENSG00000007541	0.0204	11
85	GOLGA1	ENSG00000136935	0.0127	11
86	PLTP	ENSG00000100979	0.015	11
87	MAML1	ENSG00000161021	0.0142	11
88	SOX30	ENSG00000039600	0.118	10
89	NUP160	ENSG00000030066	0.034	10
90	PLEKHG5	ENSG00000171680	0.0781	10
91	SLC26A9	ENSG00000174502	0.0371	10
92	FBLIM1	ENSG00000162458	0.0528	10
93	MRPS23	ENSG00000181610	0.0474	10
94	GPI	ENSG00000105220	0.0392	10
95	FANK1	ENSG00000203780	0.025	10
96	USB1	ENSG00000103005	0.0419	10

bioRxiv preprint doi: https://doi.org/10.1101/2025.01.08.631987; this version posted January 9, 2025. The copyright holder for this preprint (which was not certified by peer review) is the author/funder, who has granted bioRxiv a license to display the preprint in perpetuity. It is made available under aCC-BY 4.0 International license.

**97**	**PRMT7**	**ENSG00000132600**	**0.0156**	**10**
**98**	**IL4**	**ENSG00000113520**	**0.02**	**10**
**99**	**RFX2**	**ENSG00000087903**	**0.0203**	**10**
**100**	**USH1C**	**ENSG00000006611**	**0.0254**	**10**
